# High-Density Lipoprotein Reduction Differentially Modulates to Classical and Nonclassical Monocyte Subpopulations in Metabolic Syndrome Patients and in LPS-Stimulated Primary Human Monocytes *In Vitro*

**DOI:** 10.1155/2018/2737040

**Published:** 2018-04-03

**Authors:** Johanna L. Grün, Aaron N. Manjarrez-Reyna, Angélica Y. Gómez-Arauz, Sonia Leon-Cabrera, Felix Rückert, José M. Fragoso, Nallely Bueno-Hernández, Sergio Islas-Andrade, Guillermo Meléndez-Mier, Galileo Escobedo

**Affiliations:** ^1^Department of Innate Immunity and Tolerance, Institute of Transfusion Medicine and Immunology, Medical Faculty Mannheim, Heidelberg University, 68167 Mannheim, Germany; ^2^Department of Surgery, University Medical Centre Mannheim, Medical Faculty Mannheim, Heidelberg University, 68167 Mannheim, Germany; ^3^Laboratory for Liver, Pancreas and Motility, Unit of Experimental Medicine, School of Medicine, National University of Mexico, General Hospital of Mexico “Dr. Eduardo Liceaga”, 06726 Mexico City, Mexico; ^4^Laboratory for Proteomics and Metabolomics, Research Division, General Hospital of Mexico “Dr. Eduardo Liceaga”, 06726 Mexico City, Mexico; ^5^Carrera de Médico Cirujano, Unidad de Biomedicina, Facultad de Estudios Superiores-Iztacala, Universidad Nacional Autónoma de México, Avenida de los Barrios 1, 54090 Los Reyes Iztacala, MEX, Mexico; ^6^Departamento de Biología Molecular, Instituto Nacional de Cardiología “Ignacio Chávez”, Ciudad de México, Mexico

## Abstract

The effect of metabolic syndrome on human monocyte subpopulations has not yet been studied. Our main goal was to examine monocyte subpopulations in metabolic syndrome patients, while also identifying the risk factors that could directly influence these cells. Eighty-six subjects were divided into metabolic syndrome patients and controls. Monocyte subpopulations were quantified by flow cytometry, and interleukin- (IL-) 1*β* secretion levels were measured by ELISA. Primary human monocytes were cultured in low or elevated concentrations of high-density lipoprotein (HDL) and stimulated with lipopolysaccharide (LPS). The nonclassical monocyte (NCM) percentage was significantly increased in metabolic syndrome patients as compared to controls, whereas classical monocytes (CM) were reduced. Among all metabolic syndrome risk factors, HDL reduction exhibited the most important correlation with monocyte subpopulations and then was studied *in vitro*. Low HDL concentration reduced the CM percentage, whereas it increased the NCM percentage and IL-1*β* secretion in LPS-treated monocytes. The LPS effect was abolished when monocytes were cultured in elevated HDL concentrations. Concurring with *in vitro* results, IL-1*β* serum values significantly increased in metabolic syndrome patients with low HDL levels as compared to metabolic syndrome patients without HDL reduction. Our data demonstrate that HDL directly modulates monocyte subpopulations in metabolic syndrome.

## 1. Introduction

In humans, circulating monocytes have been sorted into three different subpopulations according to the cell surface expression of CD14 and CD16 [1, 2]. The vast majority of circulating monocytes show high expression of CD14 and no expression of CD16 (CD14^high^CD16^−^) and constitute the fraction of classical monocytes (CM). Monocytes producing CD16 are divided into two subgroups: intermediate monocytes (IM) that show high expression of CD14 and also show CD16 expression (CD14^high^CD16^+^) and nonclassical monocytes (NCM) that exhibit low CD14 levels accompanied by CD16 expression (CD14^low^CD16^+^) [1]. Monocyte subpopulations also show different immunological functions. The NCM subset has been shown to produce high amounts of interleukin- (IL-) 1*β* under basal conditions or in response to lipopolysaccharide (LPS) stimulation, which has led to a postulation that NCM exerts the most important inflammatory functions in circulation [3–5]. On the contrary, CM and IM subpopulations have been demonstrated to participate in endothelial adhesion and cell migration by preferably expressing chemokines and chemokine receptors without showing prominent inflammatory roles [1, 5, 6]. Thus, IL-1*β* production is considered to be a marker of inflammatory activity in human nonclassical monocytes.

Monocyte subsets are typically modulated by immune factors including tumor necrosis factor alpha (TNF-*α*) and Toll-like receptor (TLR) 2, TLR4, and TLR8 ligands [4, 7]. However, recent data show that dynamic changes in monocyte subpopulations can be influenced not only by immune agents but also by different pathophysiological conditions such as obesity [3, 4]. Indeed, a previous work of Devevre et al. has shown that an increase in body mass index (BMI) is capable of decreasing the CM percentage while also increasing the number of NCM, when comparing normal-weight controls with morbidly obese patients [3]. Interestingly, this study did not only show a direct relationship between body weight gain and increased NCM percentage but also revealed that this immune cell subpopulation seems to be the main monocytic source of IL-1*β* in obese individuals. Additional studies have consistently reported an increased percentage of NCM in subjects with BMI > 30 kg/m^2^ as compared to normal-weight individuals, which has brought to light the association between monocyte subpopulations and obesity [8]. However, it is a well-known fact that obesity is strongly linked to the development of metabolic abnormalities that are encompassed in metabolic syndrome [9]. Metabolic syndrome is a clustering of factors including abdominal obesity, hyperglycemia, high levels of blood pressure and triglycerides, and low concentration of high-density lipoproteins (HDL) that significantly increase the risk of having a cardiovascular event [10–12]. Therefore, it is reasonable to assume that metabolic syndrome may also have major effects upon monocyte subpopulations by mechanisms with the ability to alter the fragile balance among classical, intermediate, and nonclassical monocytes. Thereby, our main goal was to examine the percentages of classical, intermediate, and nonclassical monocytes in subjects with metabolic syndrome while also identifying the risk factors that could directly contribute to alter the monocyte subpopulation balance by performing *in vitro* cultures using primary human monocytes.

## 2. Materials and Methods

### 2.1. Subjects

Eighty-six women and men between 20 and 60 years old attending to the Blood Bank of the General Hospital of Mexico “Dr. Eduardo Liceaga” were included in the study. All participants provided written informed consent, previously approved by the institutional ethical committee of the General Hospital of Mexico, which guaranteed that the study was conducted in accordance with the principles described in the Helsinki Declaration. Subjects were excluded if they had previous diagnosis of diabetes mellitus, cardiovascular diseases, acute or chronic hepatic disease, acute or chronic renal disease, inflammatory or autoimmune disorders, acute or chronic infectious diseases, cancer, and endocrine disorders. We also excluded HIV-, HCV-, and HBV-seropositive patients, pregnant or lactating women, and subjects under any kind of anti-inflammatory, antiaggregant, and antihypertensive medication. All of the individuals enrolled in the study received full medical evaluation, including clinical history and physical examination by a physician.

### 2.2. Anthropometric and Metabolic Measurements

In all participants, BMI, waist circumference, and body fat percentage were recorded. The BMI is a result of dividing weight by height squared (kg/m^2^). Waist circumference was obtained from each study subject by measuring at the midpoint between the lower rib margin and the iliac crest using a conventional tape in centimeters (cm). Body fat percentage was individually recorded by means of using a body composition analyzer (TANITA® Body Composition Analyzer, Model TBF-300A, Tokyo, Japan). Systolic blood pressure was individually measured using a digital automatic blood pressure monitor (OMRON Healthcare, Germany). Blood samples were individually obtained from each subject after an 8 h overnight fasting and collected into pyrogen-free tubes (Vacutainer, BD Diagnostics, NJ, USA) at room temperature. Collection tubes were then centrifuged at 1800*g* for 10 min, and serum samples were obtained and stored at −80°C in numerous aliquots until use. Serum glucose levels were measured in triplicate by the glucose oxidase assay, following the manufacturer's instructions (Megazyme International, Ireland). Serum insulin levels were measured in triplicate by enzyme-linked immunosorbent assay (ELISA), following the manufacturer's instructions (Abnova Corporation, Taiwan). The estimate of insulin resistance was individually calculated by means of the homeostasis model assessment of insulin resistance (HOMA-IR). Total cholesterol, triglyceride, low-density lipoproteins (LDL), and high-density lipoproteins (HDL) levels were individually measured in triplicate by enzymatic assays according to the manufacturer's instructions (Roche Diagnostics, Mannheim, Germany). IL-1*β* was measured in serum samples and culture supernatants in triplicate by Sandwich ELISA (PeproTech, Mexico) using serum samples diluted 1 : 250 in PBS 1x (Sigma-Aldrich, Mexico) and culture supernatants without any dilution. All of the biochemical measurements were performed at the same time in order to avoid procedural variations.

### 2.3. Diagnosis of Metabolic Syndrome

Diagnosis of metabolic syndrome was performed according to the criteria of the National Cholesterol Education Program's Adult Treatment Panel III report (ATP III) [13], when three of five of the following factors were present: central obesity denoted by a waist circumference greater than 80 cm in women and 90 cm in men (cut-off points recommended for the Hispano-American population [14]), hypertriglyceridemia (circulating triglyceride levels > 150 mg/dL), decreased serum values of HDL-cholesterol (serum HDL < 40 mg/dL in men and 50 mg/dL in women), blood pressure higher than 120/80 mmHg, and hyperglycemia (fasting blood glucose > 100 mg/dL). According to the presence or absence of metabolic syndrome, participants were divided into metabolic syndrome and control groups.

### 2.4. White Blood Cell Isolation and Characterization of Monocyte Subpopulations by Flow Cytometry

Six milliliters of venous blood was obtained from each participant and collected into tubes containing EDTA (Vacutainer™, BD Diagnostics, NJ, USA). Collection tubes were then centrifuged at 1800*g* for 10 minutes and white blood cells (WBCs) separated using a micropipette. Total WBCs were separately placed into 1.6 mL pyrogen-free Eppendorf tubes containing 1 mL of ACK Lysing Buffer (Life Technologies, USA) and incubated at 4°C for 8 minutes. Immediately after, each sample was centrifuged at 1800g/4°C for 4 minutes and cell pellets washed twice with PBS 1x (Sigma-Aldrich, Mexico). After an additional centrifugation step and removal of the supernatant, each cell pellet was resuspended in 50 L of PBS 1x (Sigma-Aldrich, Mexico). On each test, 3 *μ*L of Human TruStrain Reagent (BioLegend Inc., USA) was added to 2 × 10^5^ WBCs and then incubated for 10 minutes on ice. Immediately after, WBCs were incubated with anti-CD14 PE/Cy7 and anti-CD16 FITC (BioLegend Inc., USA) for 30 minutes on ice for posterior analysis on a FACSCanto II flow cytometer (BD Biosciences, Mexico) by means of BD FACSDiva™ software 6.0, acquiring 1 × 10^5^ monocyte events per test in duplicate. PE/Cy7 mouse IgG2 and FITC mouse IgG1 were used as isotype control antibodies for cell surface staining of CD14 and CD16, respectively. For gating strategy, white blood cells were firstly gated for singlets on a forward scatter height (FSC-H)/forward scatter area (FSC-A) density plot. Then, lymphocyte, granulocyte, and monocyte populations were gated on a FSC-A/side scatter area (SSC-A) plot. On the monocyte gate, living cells were further gated using the Live/Dead Aqua stain (Thermo Fisher Scientific Inc., USA). Living monocytes were then gated to determine CD14- and CD16-positive expression. Assessment of monocyte subpopulations was performed according to the cell surface expression of CD14 and CD16, as follows: CD14^high^CD16^−^, classical monocytes; CD14^high^CD16^+^, intermediate monocytes; and CD14^low^CD16^+^, nonclassical monocytes.

### 2.5. HDL Removal for In Vitro Cultures

Blood samples were collected into pyrogen-free tubes (Vacutainer, BD Diagnostics, NJ, USA) from nine volunteers with diagnosis of metabolic syndrome that specifically included central obesity, fasting hyperglycemia, increased triglyceride levels, and low HDL concentration. Collection tubes were then centrifuged at 1800*g* for 10 min and serum samples obtained. Serum fractions were treated with HDL precipitation buffer (Abcam, Cambridge, MA) for HDL removal, as previously reported [15]. HDL removal was verified by Western blot using an anti-HDL polyclonal antibody that specifically recognizes a 31 kDa protein, according to the manufacturer's instructions (Abcam, Cambridge, MA). Resulting serum samples were supplemented using the same amount of RPMI 1640 medium containing 2 mM L-glutamine and 50 *μ*g/mL gentamicin (Sigma-Aldrich, Mexico), and then 0.77 mmol/L or 1.55 mmol/L purified HDL (Sigma-Aldrich, Mexico) was added. HDL-enriched culture media were differentially used to simulate the low (0.77 mmol/L = 30 mg/dL) and high (1.55 mmol/L = 60 mg/dL) HDL levels found in metabolic syndrome patients and healthy subjects, respectively. A negative control with total absence of HDL was included in all different *in vitro* culture conditions.

### 2.6. In Vitro Cultures of Primary Human Monocytes and LPS Stimulation

For *in vitro* studies, blood samples were collected into tubes containing EDTA (Vacutainer, BD Diagnostics, NJ, USA) from the same nine donors with diagnosis of metabolic syndrome mentioned above. Blood samples were separately diluted 1 : 2 with phosphate saline buffer 1x (PBS 1x, Sigma-Aldrich, Mexico) for posterior isolation of WBCs by density gradient centrifugation (Sigma-Aldrich, Mexico). Monocytes were then isolated from WBCs by CD14-positive selection using magnetic columns (Miltenyi Biotec, Germany). Purified monocytes were placed in 0.77 mmol/L or 1.55 mmol/L HDL-enriched culture media in 12-well cell-culture plates (CoStar, USA), at a density of 1 × 10^6^ monocytes per well. Each culture well was incubated with or without gram-negative bacteria-derived LPS (Sigma-Aldrich, Mexico) at 1 *μ*g/mL for six hours at 37°C in humidified 5% CO_2_ atmosphere. After 6 h incubation, LPS-stimulated and untreated monocytes were obtained and resuspended in 50 *μ*L of PBS 1x for being incubated with anti-CD14 PE/Cy7 and anti-CD16 FITC (BioLegend Inc., USA) antibodies as described above. For gating strategy, untreated and LPS-treated cells were firstly gated for singlets on a FSC-H/FSC-A density plot. On the monocyte gate, living untreated and LPS-treated cells were further gated using the Live/Dead Aqua stain. Living monocytes were then gated to determine CD14- and CD16-positive expression and identify monocyte subpopulations as follows: CD14^high^CD16^−^, classical monocytes; CD14^high^CD16^+^, intermediate monocytes; and CD14^low^CD16^+^, nonclassical monocytes. The cell viability rate was determined based on the Live/Dead Aqua stain and ranged from 91 to 96% for all acquired cell samples, without showing significant differences between untreated and LPS-treated monocytes. FACS analysis was performed in a FACSCanto II flow cytometer (BD Biosciences, Mexico) by means of BD FACSDiva software 6.0, acquiring 1 × 10^5^ monocyte events per test in duplicate. Assessment of monocyte subpopulations was performed as described above and supernatants collected for posterior IL-1*β* measuring by ELISA as mentioned before.

### 2.7. Statistical Analysis

The Shapiro-Wilk test was performed to estimate normality in data distribution and then proceed to perform Student's *t*-test to compare metabolic syndrome patients and healthy subjects in terms of BMI, waist circumference, body fat percentage, fasting glucose, fasting insulin, HOMA-IR, systolic blood pressure, total cholesterol, triglycerides, LDL, HDL, IL-1*β*, CM, IM, and NCM. Significant differences in women/men proportion were estimated by means of the chi-squared test. One-way ANOVA, followed by a post hoc Tukey test, was used to compare the serum levels of IL-1*β* in subjects with different numbers of metabolic syndrome risk factors. Data were expressed as median ± standard deviation. Pearson's correlation coefficients were calculated for examining the association of CM, IM, and NCM with anthropometric, biochemical, and immunological parameters in the study population. Pearson's correlation results were expressed as coefficients (*r*) and *P* values. Differences were considered significant when *P* < 0.05. All statistical analyses were performed using the GraphPad Prism 6.01 software.

## 3. Results

Eighty-six participants were included in the study, and no significant differences were seen between controls and subjects with metabolic syndrome in age, women/men proportion, systolic blood pressure, plasma insulin, total cholesterol, and LDL levels (Table 1). On the contrary, metabolic syndrome subjects had higher BMI than control individuals (29.92 ± 5.26 versus 26.12 ± 4.09, resp.), while waist circumference was also significantly elevated in this study group (100.41 ± 10.71 versus 90.18 ± 9.22, resp.). Furthermore, body fat percentage exhibited a significant 21% increase in metabolic syndrome subjects with respect to control individuals (33.35 ± 10.13 versus 27.38 ± 7.63 percent, resp.) (Table 1). Similarly, fasting blood glucose concentration was 30% increased in patients with metabolic syndrome as compared to controls (106.50 ± 23.48 versus 82.37 ± 18.74, resp.), while HOMA-IR value was raised by 31% (3.63 ± 1.26 versus 2.77 ± 1.21, resp.). In the same sense, triglycerides were 42% augmented in the metabolic syndrome group as compared to controls (235.53 ± 95.26 versus 165.04 ± 95.12, resp.), whereas HDL levels showed a clear 27% reduction in the scenario of metabolic syndrome with respect to normal conditions (38.53 ± 8.62 versus 53.20 ± 13.34, resp.) (Table 1). According to the ATP III criteria, the prevalence of metabolic syndrome in our study population was 58%, which suggests that half of the apparently normal subjects enrolled into the study had higher cardiovascular risk. In the metabolic syndrome group, the most frequently seen risk factor was central obesity (89%, which means that 9 in 10 metabolic syndrome patients were abdominally obese), followed by HDL reduction (78%), hypertriglyceridemia (52%), hyperglycemia (18%), and high blood pressure (13%).

**Figure 1:**
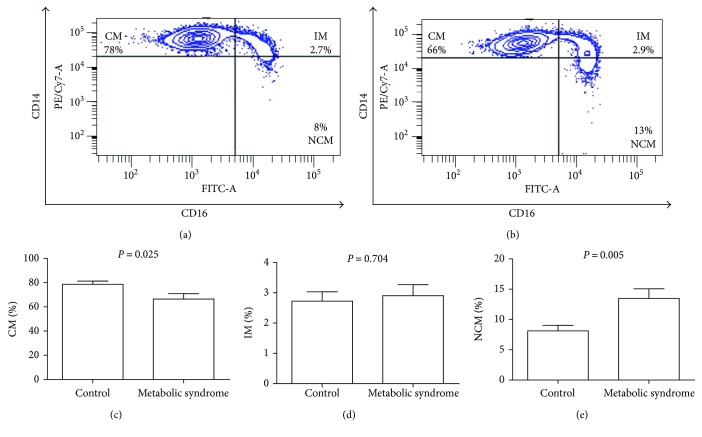
Percentage of classical, intermediate, and nonclassical monocytes in metabolic syndrome patients and control subjects. Representative flow cytometry dot plots showing the percentage of classical (CM), intermediate (IM), and nonclassical monocytes (NCM) in control subjects (a) and patients with metabolic syndrome (b). The CM percentage is significantly decreased in metabolic syndrome patients as compared to controls (c). The IM percentage showed no significant differences between metabolic syndrome patients and controls (d). The NCM percentage is significantly increased in metabolic syndrome patients as compared to controls (e). For gating strategy, white blood cells were firstly gated for singlets on a FSC-H/FSC-A density plot. Then, lymphocyte, granulocyte, and monocyte populations were gated on a FSC-A/SSC-A plot. On the monocyte gate, living cells were further gated using the Live/Dead Aqua stain. Living monocytes were then gated to determine CD14- and CD16-positive expression and identify monocyte subpopulations as follows: CD14^high^CD16^−^, classical monocytes; CD14^high^CD16^+^, intermediate monocytes; and CD14^low^CD16^+^, nonclassical monocytes. In panels (c)–(e), data are expressed as mean ± standard deviation. Significant differences were estimated by means of performing Student's *t*-test. Differences were considered significant when *P* < 0.05. Diagnosis of metabolic syndrome was performed according to the ATP III criteria, when three of five of the following factors were present: central obesity denoted by a waist circumference greater than 80 cm in women and 90 cm in men, hypertriglyceridemia (circulating triglyceride levels > 150 mg/dL), decreased serum values of HDL-cholesterol (serum HDL < 40 mg/dL in men and 50 mg/dL in women), blood pressure higher than 120/80 mmHg, and hyperglycemia (fasting blood glucose > 100 mg/dL).

Monocyte subpopulations were analyzed according to CD14 and CD16 cell surface expression by flow cytometry (Figure 1). Representative dot plots showing CD14 and CD16 expression in classical (CM), intermediate (IM), and nonclassical (NCM) monocyte subsets from control individuals and subjects with metabolic syndrome can be seen in Figures 1(a) and 1(b), respectively. The percentage of CM (CD14^high^CD16^−^) showed a significant 15% decrease in subjects with metabolic syndrome as compared to controls (66.46 ± 4.5 versus 78.62 ± 2.68, resp.) (Figure 1(c)). The IM subpopulation (CD14^high^CD16^+^) did not show any significant difference between controls and metabolic syndrome subjects (2.72 ± 0.30 versus 2.90 ± 0.36, resp.) (Figure 1(d)). In contrast, the percentage of NCM expressing CD16 and low CD14 levels exhibited a significant 65% increase in the metabolic syndrome group as compared to controls (13.47 ± 1.59 versus 8.12 ± 0.89, resp.) (Figure 1(e)).

After evaluating the percentage of classical, intermediate, and nonclassical monocyte subsets in subjects with metabolic syndrome and controls, we attempted to identify what anthropometric and metabolic variables had a significant correlation level with these innate immune cells (Table 2). In control individuals without metabolic syndrome, we saw that anthropometric parameters such as BMI, waist circumference, and body fat percentage had the strongest correlation with monocyte subpopulations (Table 2). In contrast, anthropometric parameters were shown to lose statistical correlations with monocyte subpopulations in the scenario of metabolic syndrome. However, we found a remarkable emerging association between HDL and classical and nonclassical monocytes in subjects with metabolic syndrome. In fact, a moderate relationship between increasing percentage of CM and elevated HDL levels was seen in metabolic syndrome patients (*r* = 0.531, *P* = 0.013) (Table 2). On the opposite, the NCM subpopulation showed a strong inverse association with increased concentrations of HDL (*r* = −0.621, *P* = 0.009) (Table 2). Interestingly, the inverse relationship between CM and NCM was significantly stronger when studied in patients with metabolic syndrome than in control individuals (*r* = −0.727, *P* < 0.001, and *r* = −0.585, *P* = 0.001, resp.) (Table 2).

**Figure 2:**
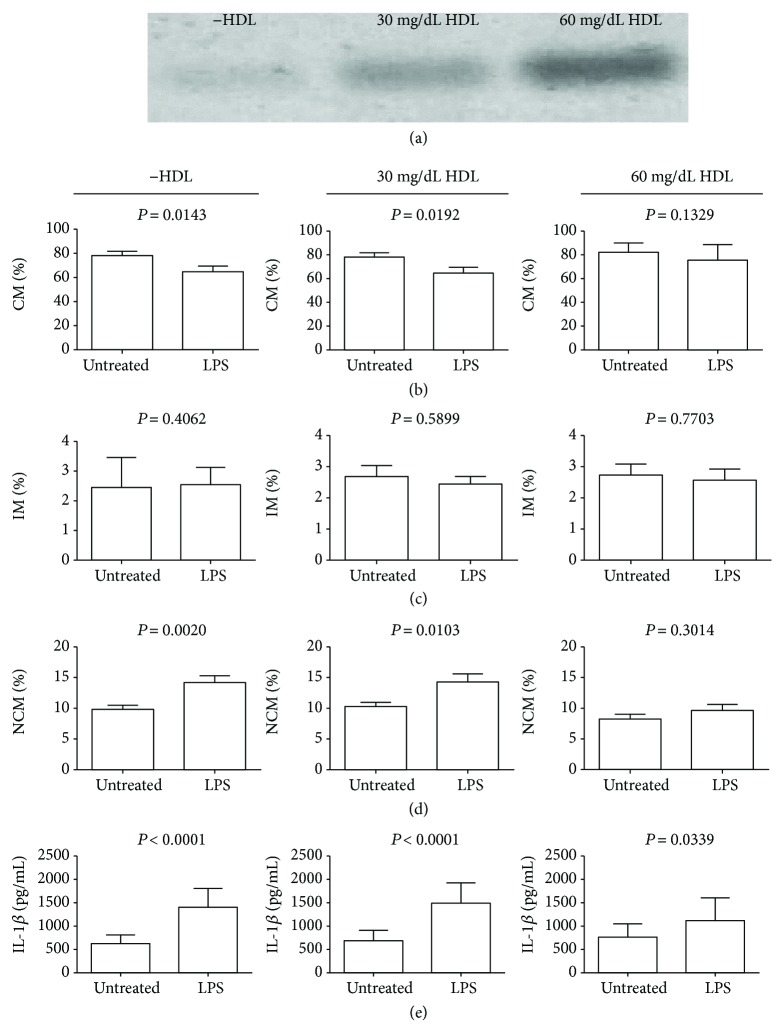
Effect of HDL on LPS-stimulated primary human monocytes *in vitro*. Representative polyacrylamide gel showing a metabolic syndrome patient's serum sample in which HDL was totally removed (−HDL) and then reconstituted with 0.77 mmol/L (30 mg/dL) or 1.55 mmol/L (60 mg/dL) HDL (a). As compared to untreated cells, LPS stimulation induced reduction in the CM percentage in low HDL levels (zero and 30 mg/dL) (b, left and middle panels, resp.). In contrast, the effect of LPS on the CM percentage was abolished in 60 mg/dL HDL that resembled a high HDL concentration (b, right panel). LPS stimulation did not significantly modify the IM percentage neither in low nor in high HDL concentrations (c, left, middle and right panels, resp.). As compared to untreated cells, LPS stimulation increased the NCM percentage in zero and 30 mg/dL HDL (d, left and middle panels, resp.). On the contrary, the effect of LPS on the NCM percentage was abolished in high HDL concentrations (d, right panel). As compared to untreated cells, LPS stimulation increased IL-1*β* production in primary human monocytes cultured in low HDL concentrations (e, left and middle panels, resp.). In contrast, the effect of LPS on IL-1*β* production was 1.5-fold reduced in 60 mg/dL HDL (e, right panel). Monocytes were isolated from white blood cells by CD14-positive selection using magnetic columns and placed in 0.77 mmol/L (30 mg/dL) or 1.55 mmol/L (60 mg/dL) HDL-enriched culture media (1 × 10^6^ monocytes per well), in the presence or absence of gram-negative bacteria-derived LPS at 1 *μ*g/mL for six hours at 37°C. After this time, monocytes were incubated with anti-CD14 PE/Cy7 and anti-CD16 FITC as described. For the gating strategy, untreated and LPS-treated cells were firstly gated for singlets on a FSC-H/FSC-A density plot. On the monocyte gate, living untreated and LPS-treated cells were further gated using the Live/Dead Aqua stain. Living monocytes were then gated to determine CD14- and CD16-positive expression and identify monocyte subpopulations as follows: CD14^high^CD16^−^, classical monocytes; CD14^high^CD16^+^, intermediate monocytes; and CD14^low^CD16^+^, nonclassical monocytes. In (b–e), data are expressed as mean ± standard deviation. Significant differences were considered when *P* < 0.05.

By means of performing statistical analyses, HDL was identified as the molecule exerting the most important association with classical and nonclassical monocytes in metabolic syndrome. For this reason, our next step was to study the effect of HDL on the monocyte subpopulation dynamic by conducting *in vitro* cell culture experiments (Figure 2). Figure 2(a) illustrates a representative polyacrylamide gel showing a metabolic syndrome patient's serum sample in which HDL was totally removed (left) and then reconstituted with 0.77 mmol/L (middle) or 1.55 mmol/L HDL (right) (Figure 2(a)). In culture conditions using low HDL levels (30 mg/dL), LPS stimulation induced a 17% reduction in the CM percentage as compared to untreated cells (Figure 2(b), middle panel). In contrast, the effect of LPS on this monocyte subset was abolished when using HDL concentrations that resembled those found in healthy subjects without metabolic syndrome (60 mg/dL) (Figure 2(b), right panel). The IM percentage tended to decrease when treated with LPS either in low HDL level or in normal HDL concentration without showing significant differences (Figure 2(c)). On the contrary, in culture conditions mimicking the HDL reduction that is observed in metabolic syndrome patients, LPS stimulation promoted a 40% increase in the NCM percentage with respect to untreated cells (Figure 2(d), middle panel). Interestingly, the LPS-stimulated NCM increase was revoked when monocytes were cultured in the presence of high HDL levels mimicking those found in healthy individuals without metabolic syndrome (Figure 2(d), right panel). Interestingly, classical and nonclassical monocytes cultured in the absence of HDL showed a similar response pattern than that found in 30 mg/dL HDL (left panels at Figures 2(b) and 2(d), resp.). As previously mentioned, IL-1*β* is a key cytokine that is mainly produced by NCM in response to LPS. In parallel to the NCM increase, LPS stimulation also enhanced IL-1*β* secretion by 216% in monocytes cultured in zero and 30 mg/dL HDL (Figure 2(e), left and middle panels, resp.). However, the effect of LPS on IL-1*β* production was 1.5-fold decreased when tested in 60 mg/dL HDL (Figure 2(e), right panel).

**Figure 3:**
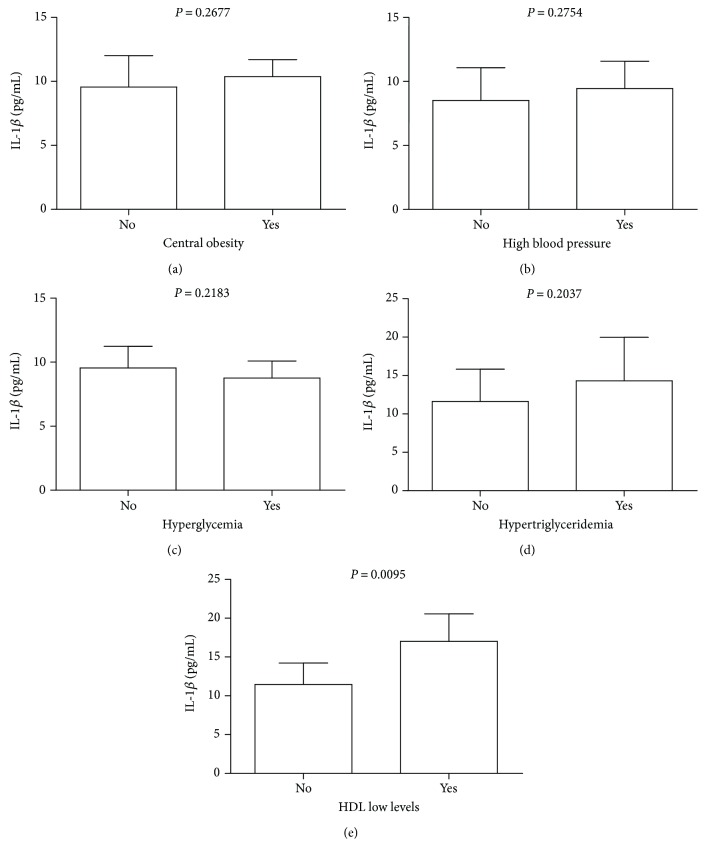
Serum levels of IL-1*β* in the study patients according to different metabolic syndrome risk factors. (a) Metabolic syndrome patients displaying central obesity (*n* = 39) showed similar IL-1*β* serum levels than did metabolic syndrome patients that had a normal waist circumference (*n* = 5). (b) The serum levels of IL-1*β* did not show significant differences in metabolic syndrome patients with elevated blood pressure (*n* = 6) as compared to metabolic syndrome patients with normal blood pressure (*n* = 38). (c) Metabolic syndrome patients showing fasting hyperglycemia (*n* = 8) exhibited similar IL-1*β* circulating levels than did metabolic syndrome patients with normal glycemic values (*n* = 36). (d) The serum levels of IL-1*β* tended to increase in metabolic syndrome patients with hypertriglyceridemia (*n* = 23) but did not show significant differences with respect to metabolic syndrome patients showing normal triglyceride values (*n* = 21). (e) In contrast, IL-1*β* was significantly increased in metabolic syndrome patients with HDL low levels (*n* = 34) as compared to metabolic syndrome patients exhibiting normal HDL values (*n* = 10). Data are expressed as mean ± standard deviation. Significant differences were estimated by means of performing the Mann–Whitney test. Differences were considered significant when *P* < 0.05. Central obesity was diagnosed when women and men had a waist circumference greater than 80 cm and 90 cm, respectively. High blood pressure was diagnosed in women and men with blood pressure values higher than 120/80 mmHg. Hyperglycemia was diagnosed in women and men with fasting blood glucose greater than 100 mg/dL. Hypertriglyceridemia was diagnosed in women and men with triglyceride values higher than 150 mg/dL. Decreased serum values of HDL were established in women and men with HDL serum values lower than 50 mg/dL and 40 mg/dL, respectively.

Since not only monocyte subpopulations were modified by HDL but also their ability to produce IL-1*β*, we decided to measure IL-1*β* serum levels in the metabolic syndrome population (Figure 3). Abdominally obese patients with metabolic syndrome showed similar IL-1*β* serum levels than those found in metabolic syndrome patients without central obesity (Figure 3(a)). No significant differences in the IL-1*β* serum levels were also seen in metabolic syndrome patients with and without high blood pressure or hyperglycemia (Figures 3(b) and 3(c), resp.). Metabolic syndrome patients with hypertriglyceridemia exhibited a nonsignificant increase in serum IL-1*β* as compared to metabolic syndrome patients without abnormally high triglyceride values (Figure 3(d)). On the opposite, the serum levels of IL-1*β* showed a significant 1.5-fold increase when studied in metabolic syndrome patients with low HDL levels as compared to metabolic syndrome patients without showing HDL reduction (Figure 3(e)).

## 4. Discussion

Previous studies have demonstrated a monocyte subset imbalance in morbidly obese patients that is mainly characterized by increased percentages of nonclassical monocytes [3]. Nevertheless, obesity is frequently accompanied by cardiovascular risk factors that are encompassed in the metabolic syndrome [10–12]. For this reason, we attempted to examine the monocyte subpopulation percentages when considering the presence of metabolic syndrome.

As expected, our data show that BMI, waist circumference, and body fat percentage are directly related to increased NCM percentage and decreased CM amount, which concurs with previous works reporting that obesity is associated with imbalance in monocyte subsets [3]. However, this association was only seen in overweight and obese subjects that did not meet the rest of criteria for being diagnosed with metabolic syndrome. Beyond obesity, in the scenario of metabolic syndrome, we found decreasing percentages of classical monocytes and prominent elevations in the nonclassical monocyte subpopulation that were related to HDL levels and showed no association with obesity-related anthropometric parameters. In this context, it is reasonable to consider the fact that the monocyte subpopulation dynamic is not only modified by obesity but is also due to other factors such as those encompassed in the metabolic syndrome, especially the serum levels of HDL.

HDL is a protein of blood plasma that is capable of binding to lipid molecules such as triglycerides and cholesterol and participates in the cholesterol clearance—the main reason why HDL is also called the “good cholesterol” [16, 17]. High serum levels of HDL have been related to low cardiovascular risk, whereas a reduction in this lipoprotein accompanied by increased levels of LDL is considered to increase the risk of having metabolic syndrome, heart disease, or stroke [16, 18]. In our study, HDL showed a positive correlation with classical monocytes but a negative relationship with nonclassical monocytes. In other words, the present results suggest that elevated HDL levels may restrict the proportion of the inflammatory nonclassical monocytes but also favor expanding the classical monocyte subset that has no prominent inflammatory actions. However, the idea that HDL seems to be associated with imbalance between classical and nonclassical monocyte subsets was conceived after performing a merely statistical approach. In view of the fact that statistical correlation models do not allow us to find a causative role for HDL in modulating monocyte subsets, we decided to culture blood-isolated monocytes in the presence of high and low HDL levels *in vitro*. To simulate the microenvironment found in metabolic syndrome, we used serum samples from subjects having HDL reduction, central obesity, hyperglycemia, and hypertriglyceridemia, in which we specifically removed HDL. Then, we deliberately supplemented each serum sample with 0.77 mmol/L (30 mg/dL) or 1.55 mmol/L (60 mg/dL) purified HDL for having monocyte culture conditions with low and high HDL levels, respectively. In this form, we were able to corroborate our *in vivo* findings by studying the effect of HDL on LPS-stimulated monocytes *in vitro*. As expected, in culture conditions simulating the microenvironment found in metabolic syndrome with low HDL levels, LPS stimulation was able to induce decreased CM percentage and at the same time increased the NCM subset, which concurs with Mukherjee et al. who reported the same result [5]. In contrast, when the HDL concentration was deliberately increased until the levels found in healthy subjects without metabolic syndrome were reached, the effect of LPS on classical and nonclassical monocytes was abolished. As mentioned, numerous studies have suggested that HDL is positively associated with classical monocytes but at the same time negatively related to nonclassical monocytes. In this sense, previous studies have demonstrated that nonclassical monocyte percentages increase as HDL plasma levels decrease in adults and children with familial hypercholesterolemia [19, 20]. Concomitantly, a positive correlation between HDL plasma levels and classical monocytes was also reported by Christensen et al. [20]. Additional studies have also confirmed that HDL levels negatively correlate with increasing percentage of NCM [21–23], which concurs with present results. Interestingly, our findings also demonstrate that absence of HDL has similar *in vitro* effects on monocyte subpopulations than those found when using 30 mg/dL HDL, which suggest that HDL may prevent raising nonclassical monocytes in concentrations above 60 mg/dL. To the best of our knowledge, this is the first report to not only suggest an association between HDL levels and monocyte subpopulations but also demonstrate that low HDL levels directly contribute to a decrease in CM and an increase in NCM in response to prototypical stimuli such as LPS. This finding brings to light the importance of studying the probable role of HDL in the regulation of the CD14 and CD16 expression in human monocytes, which is the primary feature defining classical, intermediate, and nonclassical monocyte subpopulations.

CD14 is a surface protein that preferably expresses on human monocytes and macrophages [24]. CD14 forms the trimeric LPS receptor complex together with TLR4 and MD-2, aimed at recognizing bacterial endotoxins and consequently triggering the NF*κ*B-dependent inflammatory cytokine expression such as TNF-*α*, IL-6, and IL-1*β* [24–26]. Notably, it has been reported that infusion with reconstituted HDL is able to reduce CD14 expression in peripheral monocytes of healthy volunteers while also decreasing the serum concentrations of TNF-*α* and IL-6 [27]. It is worth mentioning that monocytes isolated from reconstituted HDL-treated healthy volunteers still showed decreased expression of CD14 even upon *in vitro* stimulation with LPS [27]. Similarly, Galbois et al. demonstrated that CD14 expression is diminished in circulating monocytes of cirrhotic patients and healthy controls after having been cultured in the presence of HDL [28]. In the specific case of CD16 (an Fc*γ* receptor that binds to Fc fragments of immunoglobulin G in the cell surface of innate immune cells [1]), HDL levels have an apparent association with CD16 expression in monocytes of patients with stable coronary artery disease [21–23]. However, to the best of our knowledge, there are no experimental works studying the possible mechanisms by which HDL is linked to CD16 expression in human monocytes. Altogether, this information suggests that HDL may exert its effects on monocyte subpopulations by modulating the expression of CD14, and probably CD16, via TLR4 and NF*κ*B activation. Nevertheless, it is important to note there are not yet concluding studies aimed at characterizing the possible mechanism by which HDL is able to impact monocyte subpopulations, and the discussion of these results makes no attempt to conjecture beyond that. On the other hand, the idea that HDL exerts its effects by activating prominent inflammatory pathways could not only involve the dynamic changes found in monocyte subsets but also the ability of monocytes to produce key proinflammatory cytokines such as IL-1*β*.

IL-1*β* is a cytokine predominantly produced by nonclassical monocytes and activated macrophages with prominent roles in the regulation of the inflammatory response as well as cell differentiation and apoptosis [3, 5]. Our results show that LPS-stimulated IL-1*β* production in cultured primary human monocytes is favored when HDL is totally absent or in low levels. In other words, culture conditions mimicking the levels of HDL that are found in metabolic syndrome do not only increase the NCM percentage but also enhance production of IL-1*β*. Concurring with this notion, restoration of the HDL concentration until reaching the levels found in healthy subjects without metabolic syndrome was able to diminish the LPS-stimulated IL-1*β* production in cultured primary human monocytes *in vitro*. These findings are consistent with prior studies suggesting that high HDL values, as those seen in metabollicaly healthy individuals, seem to exert anti-inflammatory actions, whereas low HDL levels may predispose the organism to more robust proinflammatory responses [17–19, 27]. However, it is important to proceed with caution in trying to transfer our *in vitro* results that support an effect of HDL on monocyte subpopulations toward a much more complicated clinical scenario such as metabolic syndrome, in which monocytes subsets are not only in contact with HDL but also in the presence of many other risk factors. For this reason, it is still of enormous importance to clarify whether high HDL levels have prominent anti-inflammatory actions in transversal and prospective clinical trials aimed at understanding the role of HDL in metabolic syndrome and its comorbidities.

IL-1*β* has been shown to be primarily produced by nonclassical monocytes either under basal conditions or in response to LPS [3, 5]. On the contrary, classical monocytes are mainly involved in cell migration functions by predominantly expressing chemokine receptors without showing significant IL-1*β* production [5, 29–31]. Therefore, we want to speculate that nonclassical monocytes were the main cellular source of IL-1*β* in our *in vitro* experiments, a notion that concurs with the fact that while this monocyte subpopulation increased the IL-1*β* production also augmented and both of them were downregulated by HDL. These *in vitro* findings agree with our *in vivo* results showing that serum values of IL-1*β* significantly increased in metabolic syndrome patients with low HDL levels as compared to metabolic syndrome patients without HDL reduction. Thus, present data suggest a direct relationship among increased percentage of nonclassical monocytes, elevated concentrations of IL-1*β*, and low HDL levels in patients with metabolic syndrome. In this sense, a previous work reported an association between IL-*β* gene polymorphisms and metabolic syndrome in patients with coronary heart disease [32]. Furthermore, Al-Shorman et al. recently showed that IL-1*β* serum levels are significantly elevated in obese adolescents with metabolic syndrome as compared to normal-weight controls without metabolic alterations [33]. Altogether, this information concurs with our data that demonstrate elevation in the serum levels of IL-1*β* in patients with metabolic syndrome. However, to the best of our knowledge this is the first report suggesting that nonclassical monocytes could be a main cellular source of IL-1*β* in subjects with metabolic syndrome that show low HDL levels. The idea that both nonclassical monocytes and IL-1*β* may exert their prominent inflammatory actions in metabolic syndrome patients is congruent with numerous works showing that systemic inflammation is linked to the pathogenesis of metabolic dysfunction [7, 18, 34, 35]. However, IL-1*β* was only measured in serum samples and direct evidence regarding the role of nonclassical monocytes in IL-1*β* production in metabolic syndrome patients remains to be further elucidated; therefore, discussion of these results makes no attempt to conjecture beyond that.

A final phenomenon captured in our study is that both classical and nonclassical monocytes show a strong interdependent relationship, most of all in the setting of metabolic syndrome. It has been previously demonstrated that M1 macrophages can be shifted toward M2 macrophages, and vice versa, in response to the cell microenvironment [36–38]. However, there is scant information supporting a possible shift between monocytes with no prominent inflammatory actions and monocytes primed to inflammatory activities. In this sense, our results suggest that classical monocytes may be converted into nonclassical monocytes in response to metabolic syndrome risk factors such as HDL, thus supporting the notion that monocytes and macrophages may be primed toward proinflammatory activation profiles in the early stages of metabolic dysfunction. The possibility that monocyte subsets can be influenced by metabolic syndrome risk factors should be taken into consideration when designing molecular therapeutic interventions aimed at switching nonclassical monocytes into classical monocytes in patients at higher cardiovascular risk such as individuals with metabolic syndrome.

## 5. Conclusions

Our data demonstrate for the first time that HDL reduction directly contributes to an increase in the nonclassical monocyte subpopulation and concomitantly a decrease in the classical monocyte percentage in patients with metabolic syndrome and in LPS-stimulated primary human monocytes *in vitro*. In this work, HDL reduction was also shown to induce higher IL-1*β* production in LPS-stimulated primary human monocytes and associate with increased IL-1*β* serum levels in patients with metabolic syndrome. Altogether, these findings support the notion that metabolic dysfunction has a pivotal component in the systemic inflammatory response that is mediated by dynamic changes in monocyte subpopulations. The exact molecular mechanisms by which HDL is able to modulate monocyte subpopulations and IL-1*β* production remain to be elucidated. The potential impact of understanding the role of metabolic signals in immune cell activation adds a compelling degree of urgency to further studies.

## Figures and Tables

**Table 1:** Demographical and metabolic parameters of the study population.

Parameters	Control	Metabolic syndrome	*P* value
Gender (W/M)	17/25	16/28	0.313
Age (years)	49.25 ± 5.88	48.38 ± 5.47	0.296
BMI (kg/m^2^)	26.12 ± 4.09	29.92 ± 5.26	0.006
Waist circumference (cm)	90.18 ± 9.22	100.41 ± 10.71	0.004
Body fat (%)	27.38 ± 7.63	33.35 ± 10.13	0.012
SBP (mmHg)	124.0 ± 2.47	126.0 ± 5.61	0.306
FBG (mg/dL)	82.37 ± 18.74	106.50 ± 23.48	0.001
Insulin (mU/L)	13.67 ± 5.30	13.90 ± 3.82	0.428
HOMA-IR	2.77 ± 1.21	3.63 ± 1.26	0.005
Total cholesterol (mg/dL)	209.04 ± 41.49	200.26 ± 32.87	0.204
Triglycerides (mg/dL)	165.04 ± 95.12	235.53 ± 95.26	0.006
HDL (mg/dL)	53.20 ± 13.34	38.53 ± 8.62	0.001
LDL (mg/dL)	116.95 ± 33.45	109.50 ± 29.46	0.203

Data are expressed as mean ± standard deviation. The Shapiro-Wilk test was used to estimate normality in data distribution. Significant differences were estimated by means of performing Student's *t*-test with the exception of women/men proportion in each group, which was estimated by means of the chi-squared test. Differences were considered significant when *P* < 0.05. W: women; M: men; BMI: body mass index; SBP: systolic blood pressure; FBG: fasting blood glucose; HOMA-IR: homeostatic model assessment of insulin resistance; HDL: high-density lipoprotein; LDL: low-density lipoprotein. Diagnosis of metabolic syndrome was performed according to the ATP III criteria, when three of five of the following factors were present: central obesity denoted by a waist circumference greater than 80 cm in women and 90 cm in men, circulating triglyceride levels > 150 mg/dL, serum HDL < 40 mg/dL in men and 50 mg/dL in women, blood pressure higher than 120/80 mmHg, fasting blood glucose > 100 mg/dL.

**Table 2:** Correlation coefficients of monocyte subpopulations with anthropometric, metabolic, and cellular parameters in patients with metabolic syndrome and controls.

Parameters	Control						Metabolic syndrome					
	CM		IM		NCM		CM		IM		NCM	
	*r*	*P*	*r*	*P*	*r*	*P*	*r*	*P*	*r*	*P*	*r*	*P*
Age	0.242	0.126	0.046	0.414	−0.217	0.153	0.078	0.351	0.203	0.159	−0.028	0.446
BMI	*−0.523*	*0.004*	0.140	0.256	*0.592*	*0.001*	0.037	0.427	−0.077	0.354	0.034	0.433
WC	−0.323	0.065	0.220	0.155	*0.353*	*0.049*	0.103	0.307	0.011	0.477	−0.011	0.477
Body fat	*−0.363*	*0.043*	0.236	0.139	*0.438*	*0.018*	0.240	0.118	−0.005	0.488	−0.051	0.402
SBP	0.024	0.548	0.017	0.471	0.057	0.210	0.051	0.412	0.072	0.347	0.054	0.207
FBG	−0.237	0.132	−0.342	0.059	0.326	0.081	0.139	0.249	0.150	0.231	−0.082	0.344
Insulin	−0.012	0.476	−0.006	0.487	−0.052	0.403	−0.066	0.373	0.103	0.306	0.025	0.451
HOMA-IR	−0.042	0.421	−0.161	0.224	0.098	0.323	0.017	0.465	0.172	0.199	−0.005	0.489
Cholesterol	−0.204	0.169	0.171	0.212	−0.093	0.332	0.010	0.478	0.008	0.484	−0.175	0.196
Triglycerides	−0.111	0.306	−0.018	0.466	−0.187	0.196	−0.009	0.481	0.185	0.181	−0.154	0.226
HDL	−0.032	0.440	−0.180	0.198	0.086	0.343	*0.531*	*0.013*	0.209	0.152	*−0.621*	*0.009*
LDL	−0.214	0.156	0.249	0.120	−0.046	0.414	−0.144	0.240	−0.158	0.220	0.026	0.449
CM	—	—	0.325	0.060	*−0.585*	*0.001*	—	—	0.237	0.121	*−0.727*	*<0.001*
IM	0.319	0.064	—	—	0.084	0.695	0.237	0.121	—	—	−0.166	0.207
NCM	*−0.585*	*0.001*	0.084	0.347	—	—	*−0.727*	*<0.001*	−0.166	0.207	—	—

Coefficients (*r*) and *P* values were calculated by Pearson's correlation model. The correlation level was considered significant when *P* < 0.05. Significant associations are marked in italics. CM: classical monocytes; IM: intermediate monocytes; NCM: nonclassical monocytes; BMI: body mass index; WC: waist circumference; SBP: systolic blood pressure; FBG: fasting blood glucose; HOMA-IR: homeostatic model assessment of insulin resistance; HDL: high-density lipoprotein; LDL: low-density lipoprotein. Diagnosis of metabolic syndrome was performed according to the ATP III criteria, when three of five of the following factors were present: central obesity denoted by a waist circumference greater than 80 cm in women and 90 cm in men, hypertriglyceridemia (circulating triglyceride levels > 150 mg/dL), decreased serum values of HDL-cholesterol (serum HDL < 40 mg/dL in men and 50 mg/dL in women), blood pressure higher than 120/80 mmHg, and hyperglycemia (fasting blood glucose > 100 mg/dL).
